# Patterns of Growth Costs and Nitrogen Acquisition in *Cytisus striatus* (Hill) Rothm. and *Cytisus balansae* (Boiss.) Ball are Mediated by Sources of Inorganic N

**DOI:** 10.3390/plants5020020

**Published:** 2016-04-16

**Authors:** María Pérez-Fernández, Elena Calvo-Magro, Irene Ramírez-Rojas, Laura Moreno-Gallardo, Valentine Alexander

**Affiliations:** 1 Department of Physical, Chemical and Natural Systems, University Pablo de Olavide, Carretera de Utrera Km, Seville 141013, Spain; ecmagro79@gmail.com (E.C.-M.); iramroj@alu.upo.es (I.R.-R.); lmorgal@alu.upo.es (L.M.-G.); 2 Botany and Zoology Department, University of Stellenbosch, Private Bag X1, Matieland 7602, South Africa; alexvalentine@mac.com

**Keywords:** legume, N_2_ fixation, mineral N, C construction costs

## Abstract

Nitrogen-fixing shrubby legumes in the Mediterranean area partly overcome nutrient limitations by making use of soil N and atmospheric N_2_ sources. Their ability to switch between different sources lets them adjust to the carbon costs pertaining to N acquisition throughout the year. We investigated the utilization of different inorganic N sources by *Cytisus balansae* and *Cytisus striatus*, shrubby legumes under low and a sufficient (5 and 500 µM P, respectively) levels of P. Plants grew in sterile sand, supplied with N-free nutrient solution and inoculated with effective *Bradyrhizobium* strains; other treatments consisted of plants treated with (i) 500 µM NH_4_NO_3_; and (ii) 500 µM NH_4_NO_3_ and inoculation with effective rhizobial strains. The application of NH_4_NO_3_ always resulted in greater dry biomass production. Carbon construction costs were higher in plants that were supplied with mineral and symbiotic N sources and always greater in the endemic *C. striatus*. Photosynthetic rates were similar in plants treated with different sources of N although differences were observed between the two species. Non-fertilized inoculated plants showed a neat dependence on N_2_ fixation and had more effective root nodules. Results accounted for the distribution of the two species with regards to their ability to use different N sources.

## 1. Introduction

In natural stands where trees are absent, shrubby leguminous species play an important role in sustaining stand productivity and environmental values by regulating water uptake, the root environment and nutrient cycling [1,2]. This is of particular relevance in arid areas of the world, where nutrients are impoverished and plant growth is strongly hindered by limiting factors such as water, extreme temperatures and excessive solar radiation [3,4]. In such ecosystems, legumes contribute to global fertility by introducing nitrogen to the soils via their nitrogen-fixing symbiosis with legume-nodulating bacteria (for a review see [5,6]).

Legumes are the entryway through which nitrogen (N) enters ecosystems [6]. This provides legumes with a complementary N source as compared to non-legumes. However, little is known about the plant’s preferences with respect to N sources. It has been proven that the Cape Fynbos legume, *Virgilia divaricata* (Adamson), is able to switch N sources for its growth, depending on the environmental cues [7]. Accordingly, Neff *et al*. [8] suggested that leguminous plants can absorb and assimilate nitrogenous compounds such as nitrate, ammonium, or amino acids directly from soil in response to both the plant’s needs and the environmental restrictions imposed on organic matter decomposition. Changes in the sources of nitrogen are expected to induce differing responses in different plants species that would translate into contrasting photosynthetic rates and biomass production [9,10].

Biological nitrogen fixation (BNF) is not free and the plant must contribute a significant amount of energy in the form of photosynthates (photosynthesis-derived sugars) and other nutritional factors for the bacteria. However, some legumes are more efficient than others in fixing nitrogen. The process requires 160 Kcal·mol^−1^ for a molecule of N_2_ to be reduced [11,12]; hence, plants would only become involved in such a reaction when there are no sources of nitrogen other than the atmosphere [13].

The quantity of nitrogen fixed depends, amongst other factors, on the level of soil nitrogen, the rhizobia strain infecting the legume, the amount of legume plant growth, and the length of the growing season. Increased soil nitrogen availability results in decreased nodulation rates and N-fixing efficiency [14,15,16,17]. If given a choice, a legume plant will remove nitrogen from the soil before obtaining nitrogen from the air through N_2_-fixation, thus reducing the benefits of the nodulation. A legume growing on a sandy soil, very low in nitrogen, will get most of its nitrogen from the air while a legume growing on a fertile river-bottom soil will get most of its nitrogen from the soil [18,19].

In the central western area of the Iberian Peninsula, water is the primary factor that limits plant growth. In addition to water scarcity, soils in the best part of this area are infertile due to deep soil erosion that drives losses of N, phosphorus (P) and other nutrients that hinder plant establishment [20]. At the same time, soils are characterized by low pH values, which are known to reduce the ability of legumes to establish effective symbiosis with their rhizobial symbionts, hence reducing BNF [21,22].

These soils typically harbor low concentrations of N and P, in amounts that are generally available for plant use in micro-molar concentrations, compromising metabolic process [23]. The proportion of N:P and co-limitation are important in explaining N-P relationships in plants, and can be used as a tool to diagnose both plant growth and dynamics with respect to nutrient availability in soils [24]. Legumes are highly dependent on the P concentration in the growing media in terms of nodulation and BNF [25,26,27]. P micro-molar concentrations are extremely low to drive the P-requiring metabolic processes [19], compromising the wellbeing of plants; however, different species may response differently to N:P ratio changes under altered growing conditions, which may then account for the species distribution with regards to nutrient availability.

*Cytisus striatus* (Hill) Rothm. is a shrubby legume endemic to the Iberian Peninsula that has colonized other parts of the world [28]. It grows in siliceous soils from 450–750 m a.s.l., avoiding cold distributions. In ecotonal areas, it can form loose mixed population with *Cytisus balansae* (Boiss.) Ball. The latter is well represented in the Iberian Peninsula and northern Morocco, forming dense populations on siliceous soils from 750–1300 m a.s.l. Both *C. balansae* and *C. striatus* have been reported to nodulate with *Bradyrhizobium* spp. [2,29,30]. The main objective of this work is to identify possible ways in which inorganic N is used by *C. balansae* and *C. striatus* in relation to P availability, and how it affects carbon construction costs, photosynthetic rates and efficiency of N-fixing in these two shrubs. Our working hypothesis is that the distribution of these two species in soils with low concentrations of nutrients, under harsh climatic conditions, is explained by the plants’ ability to change their sources of N, either from the atmosphere or from the soil, during growth. Should this hypothesis be proven, it would be possible (i) to explain why these two species do not form mixed populations and (ii) to relate *C. striatus*’ ability to colonize new areas to its greater plasticity in terms of N use under low construction costs.

## 2. Materials and Methods

### 2.1. Plant Material and Experimental Design

Seeds of *C. balansae* (cba) and *C. striatus* (cst) were hand harvested in the summer of 2014 from natural populations in central-west Spain. The strains cba and cst had been previously obtained from nodules of *C. balansae* and *C. striatus* plants in monospecific natural populations [29]. Strains were identified as *Bradyrhizobium* isolates with the accession numbers AF461191 and AF461194 for cba and cst, respectively. The strains were maintained on yeast extract mannitol (YEM) agar [31] at 4 °C. For inoculation of seedlings, cultures were grown for 6 days in YEM broth at 26 °C in an orbital shaker at 100 rpm before dilution to the required concentration of cells.

Seeds were hand scarified using an emery board. This treatment was followed by surface-sterilization in 70% ethanol for 5 min and 1% sodium hypochlorite for 3 min and then washed six times in sterile distilled water. Twenty-five seedlings per species were transplanted to 10-cm diameter pots containing sterile sand-river and were given the appropriate treatment (all seedlings were supplied with 25% Hoagland’s solution—pH 5.8) [32], modified with either high P (500 μM) or low P (5 μM) as NaH_2_PO_4_ 2H_2_O). Plants were maintained in a glasshouse at the University Pablo de Olavide (Seville, Spain) under natural light and temperature, with a 12-h photoperiod (24 °C day and 18 °C night) and a photon flux density at the top of the plants of approximately 700 μmol ·m^−2^·s^−1^ for 22 weeks (February until July 2015). Pots with different treatments were randomly distributed on benches in the glass house, 1 m apart from any other treatment, to prevent cross contamination; a total of 25 replicates per combination of species and treatments were maintained.

The control treatment consisted of un-inoculated Hoagland’s solution from which nitrogenous compounds had been removed (−N−R). One of the treatments consisted of nitrogen-free Hoagland’s solution and rhizobial inoculation (−N+R). A second treatment consisted of the application of 500 μM NH_4_NO_3_ as an N source with no rhizobial inoculation (+N−R). In the last experiment, plants received the same amount of NH_4_NO_3_ as before and were simultaneously inoculated (+N+R). All treatments were subjected to both high and low P levels.

Inoculation treatments consisted of growth phase broth-cultured inoculant at 1 × 10^8^ cells mL^−1^. Each plant species was inoculated with 100 mL of its own rhizobia, *i.e.*, cba (AF461191) and cst (AF461194). The surface of the pots was covered with sterile polyurethane beds and watering was conducted weekly through a watering pipe.

### 2.2. Harvesting and Nutrient Analysis

At harvest, plants were assessed for root nodule number, shoot and root dry matter, total nitrogen accumulation in shoots and biologically-fixed nitrogen (δ^15^N). The dry mass of shoot, root and nodules was obtained as the dry weight of plant material after drying in an oven at 50 °C for 48 h–72 h. The dried material was ground and analyzed for C, N and P concentrations. The nitrogen accumulated in shoots was calculated by multiplying the weight of dry shoots by the nitrogen content as measured by the semi micro-Kjeldahl method [33]. Milled dry shoots were sent for isotopic analysis to the UIB (University of the Balearic Islands, Balearic Islands, Spain) and for total N analyses to the Laboratório Químico Agrícola Rebelo da Silva (Lisbon, Portugal).

### 2.3. Calculations of %Ndfa

The isotopic ratio of δ^15^N was calculated as δ = 1000‰ (Rsample/Rstandard), where *R* is the molar ratio of the heavier to the lighter isotope of the samples and standards are defined by [34].

The fraction of N derived entirely from N_2_ fixation (Ndfa) in the nodulated plants [35] was calculated as:

%Ndfa = (δ^15^Nreference plant − δ^15^Nlegume)/(δ^15^Nreference plant − B) × 100

where: δ^15^Nref— is the δ^15^N from a non-fixing N_2_ reference plant (*Lolium perenne* in this study); B is the δ^15^N natural abundance of the N derived from biological N-fixation of the above-ground tissue of *C. balansae* and *C. striatus*, grown in an N-free culture (plants only N source was N_2_). The B value of *C. balansae* was determined in this study as −3.94‰ and that of *C. striatus* was −2.96‰. The total amount of N in the plant derived from N_2_ fixation (*Nfix*) was determined as N*fix* = Ndfa × N content.

### 2.4. Carbon and Nutrition Cost Calculations

Construction costs, C_W_ (mmolCg^−1^DW), were calculated according to the methods proposed by [36], modified from the equation used by [37]:

C_W_ = (C + kN/14 × 180/24) × (1/0.89) × (6000/180)

where C_W_ is the construction cost of the tissue (mmolCg^−1^DW), C is the carbon concentration (mmolCg^−1^), k is the reduction state of the N substrate (k = + 5 for NO_3_) and N is the organic nitrogen content of the tissue (g^−1^DW) [38]. The constant (1/0.89) represents the fraction of the construction costs that provide reductant that is not incorporated into the biomass [37,38] and (6000/180) converts units of g glucose DW^−1^ to mmolCg^−1^DW.

Belowground allocation is the fraction of new biomass formed in terms of roots and nodules over the growth period. This was calculated according to [39]:

*df/dt* = RGR × (∂ − B_r_/B_t_)

RGR is the relative growth rate (mg·g^−1^·day^−1^) and ∂ is the fraction of new biomass gained during the growth period. B_r_/B_t_ is the root weight ratio, based on total plant biomass (B_t_) and root biomass (B_r_).

### 2.5. Photosynthetic Rates

Photosynthesis was measured using a Licor 6200 Photosynthetic System (LICOR, Lincoln, NE, USA), equipped with a quarter-liter chamber. Measurements were made between 08:00 and 16:30 h when light quality was optimum in the growing area. As leaves of the study species are particularly small, full branches (also photosynthetically active) were enclosed in the chamber. Ten measurements were performed per treatment. Light during the measurements remained steady at saturation (±1400 mol·m^−2^·s^−1^) at photosynthetic biomass temperature of 24 °C and humidity level of about 40%–60%. Surface area of photosynthetically active parts was measured using a Licor 3000 leaf area meter (LICOR). Branches were dried to constant mass and weighed for calculating leaf mass per area (LMA, g nr^2^).

### 2.6. Statistical Analysis

All data were tested for normality and homogeneity of variances using the Levene and Cochran tests. The effects of the factors and their interactions were tested using analysis of variance (ANOVA). When the ANOVA results revealed significant differences between treatments, the means (6–8) were separated using a *post^-^hoc* *t*-Student test (*p* ≤ 0.05). Statistical analysis was computed using the SPSS software version 15.0 for Windows.

## 3. Results

### 3.1. Biomass Production

High seedling mortality was observed in the −N−R treatment both at low and high P levels; the remaining seedlings had yellow leaves showing the lack of nitrogen nutrition and poor biomass production. Plants of the two species grown at the high level of phosphate always had greater biomass production in all treatments, except for those in the −N−R. The addition of NH_4_NO_3_ (+N treatments) always triggered biomass accumulation (Table 1). Under the two levels of phosphate, the application of selected inoculants (+R treatments) resulted in increased biomass production compared with mass accumulation in the control plants. There was a differential biomass production in the +R treatments under the low and high levels of P. When P was scarce, the simultaneous addition of chemical N and inoculation significantly increased biomass production. Under high phosphate, rhizobial inoculation with or without mineral-supplied N induced a biomass decrease, especially for cst compared with plants only supplied with N (Table 1).

### 3.2. Carbon Construction Cost and Photosynthetic Rate

**Figure 1:**
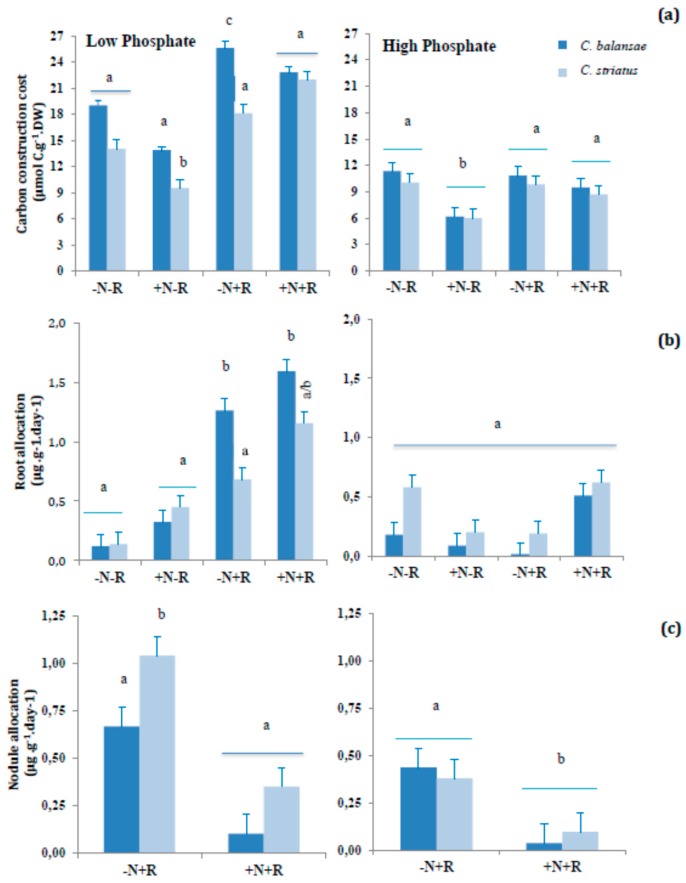
(**a**) Plant construction costs; (**b**) Root allocation and (**c**) Nodule allocation of 22-week-old *Cytisus balansae* (cba) and *Cytisus striatus* (cst) seedlings, grown in sand culture treated with −N−R, +N−R, +N−R and +N+R, under two levels of phosphate (Low and High). Values are means (*n* = 10, except for −N−R where *n* = 6) ± standard deviation. Different letters indicate significant differences among treatments (* *p* ≤ 0.05).

There were significant differences in values of carbon construction costs between plant species and treatments. *C. balansae* always showed greater carbon costs than *C. striatus*. Carbon construction costs for the two plants species were significantly greater at the low phosphate level, with the greatest values in the presence of inoculants (+R treatments) (Figure 1a). Inoculated plants supplied with N in the low P study showed the lowest C construction cost, in contrast to those with no nitrogen added. Despite the high carbon construction costs in the +N+R treatment, both cba and cst resulted in the greatest amounts of biomass (Table 1). In addition, the greatest biomass production in *C. striatus* at the high P level was achieved under the treatment +N−R that is the one for which plants showed the greatest C construction cost. At the high level of P, no differences in carbon construction costs were detected in the species except for those under the +N−R treatment, which were significantly lower (Table 1 and Figure 1a). Under the low P level, the two species’ allocation of resources to the roots was less pronounced in plants grown under the −N−R treatment. When the level of P was high, cst showed significantly greater root allocation in treatments −N−R and +N+R (Figure 1b). Nodule allocation was higher for the inoculated plants that relied solely on N_2_ fixation, compared with plants with combined N sources (Figure 1c). Under low P, nodule allocation of cst was significantly greater than that of cba; however, under the high P conditions, no statistical differences in nodule allocation were observed between cba and cst (Figure 1c). The photosynthetic rate was not influenced by any of the treatments nor by the P levels (Table 2) except for the plants in treatment −N−R. Under the high level of P, the photosynthetic rate was always greater for plants in any of the four treatments, showing a clear positive effect of this nutrient on plant performance (Table 2).

### 3.3. Nitrogen Fixation

**Figure 2:**
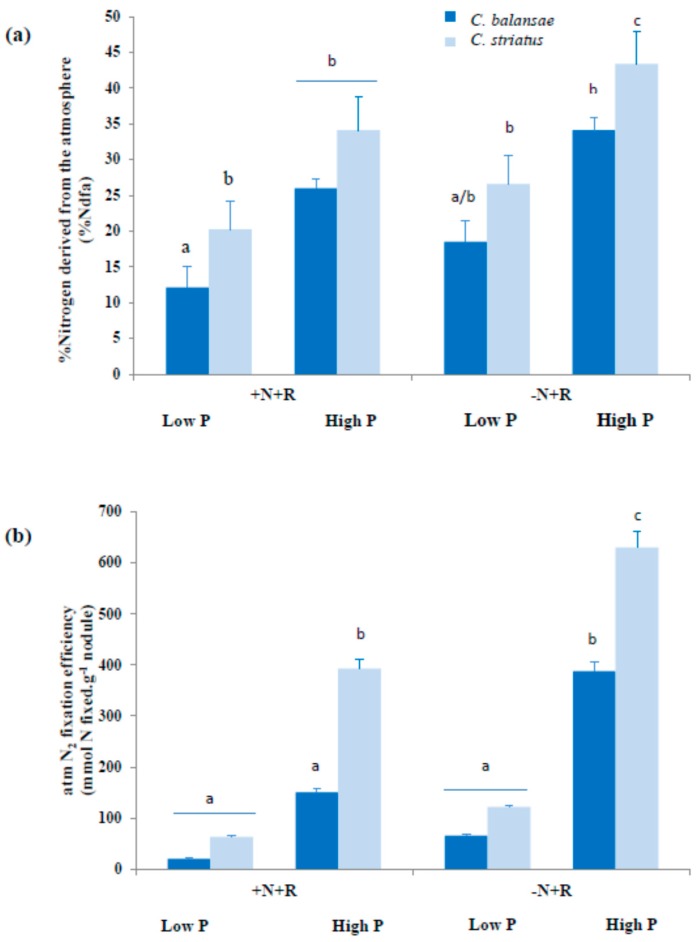
(**a**) Percentage N derived from the atmosphere (%Ndfa) and (**b**) N_2_ fixation efficiency of 22-week-old *Cytisus balansae* (cba) and *Cytisus striatus* (cst) seedlings, grown in sand culture treated with −N−R, +N−R, +N−R and +N+R, under two levels of phosphate (Low and High). Values are means (*n* = 10, except for −N−R where *n* = 6) ± standard deviation. Different letters indicate significant differences among treatments (* *p* ≤ 0.05).

Total N_2_ fixation varied between species and amongst treatments. N_2_ was significantly lower in cba than in cst; overall for the two species, N_2_ fixation efficiency was greater in the high P treatment than in the low P treatment (Figure 2a). The amount of N_2_ fixed biologically was significantly lower in plants supplied with NH_4_NO_3_, as indicated by the decline in %Ndfa in plants in the +N+R treatment compared with plants grown in the inoculated treatment (−N+R) (Figure 2a). With the exception of the −N−R treatment, for which N concentration was significantly low (1.07 ± 0.06; *p* = 0.038), there were no differences for this variable in the +N−R (1.91 ± 0.23; *p* < 0.05), −N+R (2.08 ± 0.09; *p* < 0.043) and +N+R (2.46 ± 0.11; *p* < 0.021) treatments. N_2_ fixation efficiency was greater in cst than in cba. Plants solely reliant on N_2_ fixation were more efficient at fixing N at the two levels of P according to the amounts of N fixed per nodule (Figure 2b).

## 4. Discussion

Legumes are able to change the sources of N they use to meet their metabolic needs [40,41]. The two studied species in the present research confirm this fact, and the reported behavior in terms of N use matches their current distribution in nature. Under control conditions, we tested the responses of *C. balansae* and *C. striatus* to changes in P and N supplies as well as the role of rhizobial inoculation in plant growth and biomass allocation. Both species show shifts from organic to inorganic forms of N when P in the growing media is present, which allows them to adjust to changing environmental conditions. Strong differences in plant performance under −N+R, +N−R, +N+R with significantly greater biomass production compared with plants under the −N−R treatment prove the strong dependence of the two species on N and P availability. Similarly, the dependence of both species on N was clearly moderated by the micro-molar concentrations of P in the growing media. 

Under sufficient levels of phosphorus in the growing media, both *C. balansae* and *C. striatus* were more efficient at incorporating NH_4_NO_3_ than at fixing atmospheric N. This can be explained by the fact that it is less expensive to acquire mineral sources of N than to fix them from the atmosphere [42]. The immediate result is a noticeable increase in biomass production when N and P are sufficient in the media. That would translate into a profuse colonization of soils by either of the two species. However, not all species are likely to colonize all soils because the amounts of nutrients needed for satisfactory plant growth would vary from one species to another. Differences in nutritional needs are linked to the legume-rhizobia combination as well as the inorganic source of N [42,43]. Similarly, legumes differ in their P requirements and in their ability to assimilate P from the soil [44,45], which correlates with their colonization status [46]. Most legumes from Western Australia would be killed by P concentration in soils from the Iberian Peninsula (toxic effect), whereas the latter would show P deficiencies if grown in the P-impoverished soils from Western Australia [47]. In our study, plants of cst grown under low P produced greater amounts of biomass and fixed more N_2_ than those of cba. As all plants from the two species were experimentally maintained under exactly the same glasshouse conditions and nutrient availability, we explain the greater biomass production by cst in terms of greater efficiency of the legume-rhizobia interaction [42], which can simultaneously explain its ability to effectively colonize soils beyond its natural area of distribution [48,49]. It has been demonstrated that legumes under low or zero concentrations of P and N are forced to acquire N through symbiotic N_2_ fixation; on the other hand, when N is present in the soil, legumes avoid the expensive process of N_2_ reduction [50,51]; this very same scheme is depicted by cba and cst in this experiment, which resembles the behavior of the Fynbos legume *Vigiglia divaricata* [7]. Nevertheless shifts in the use of N are actually mediated by levels of P. Under limiting levels of P for plant growth, both atmospheric N_2_ and NH_4_NO_3_ supplies contribute to increase the C sink strength of cba and cst plants in order to maintain enough carbon in the plant tissues to maintain both N fixation and soil N acquisition. Concomitantly, carbon costs and root allocations of plants in the +N+R treatments were the greatest amongst all treatments; that fact proves that the plants of the two species have to maintain the structures for N acquisition [7,50,51]. We observed differences in plant biomass accumulation under low P and N supply between the two species, with a marked biomass production by cba, which we explain in terms of carbon sink strength and lower photosynthetic rate of *C. balansae*. This behavior, which might have to be related to evolutionary processes through which cba selectively occupies areas with limited resources, also accounts for the restricted distribution of this species and the extended distribution of *C. striatus* [48]. It is interesting that plants under the +N+R and −N+R treatments showed the greatest values of C construction costs regardless of the level of P they were supplied with. Plants in these treatments also showed the greatest leaf area per plant mass. This can only be interpreted as a way to increase the leaf area ratio that the two species need to meet for the photosynthetic requirements to build up the nodules, as has been demonstrated in *Glycine max* (L. Merr.) [52] and *Virgilia divaricata* [7].

An external supply of mineral N exerts inhibiting effects on nodulation and nitrogen fixation [13,42], which are dependent on the combination of plant-rhizobia and seem to be driven by the bacterial strains [42,53]. This was clearly shown by *C. balansae* and *C. striatus* in our experiment, where a reduction in %Ndfa was observed in plants supplied with the combined sources of N; the plant species that achieved the greatest efficiency in the use of different sources of N was cst, which has allowed this species to expand its area of distribution. The two Iberian shrubs have shown behavior similar to that previously described for *V. divaricata* [7], *i.e.*, a decrease in %Ndfa when plants have enough P and inorganic sources of N, regardless of the presence of inoculants in the media This proves that plants tend to use less energy demanding sources of N (mineral sources).

Overall, these results support the initial hypothesis that the ability of the shrubby legume *C. striatus* to change sources of N plays a role in its distribution and that limitations of *C. balansae* to efficiently fix N_2_ has restricted its distribution. Similarly, the broader tolerance of cst to P and N concentrations in the soil account for its extended distribution. We have shown that *C. striatus* is the species that can make better use of any available source of N and at the time, is the one with the lowest carbon costs (at a constant photosynthetic rate). *C. balansae* plants are more reliant on inorganic sources of N, and the maintenance of nodules corresponds to the greatest carbon constructions costs, which represents a strong limit to its growth. Differences in the behavior of the two species and restricted ability to quickly and efficiency change the use of N might be the reasons why *C. striatus* continues to expand and *C. balansae* is restricted in its area of distribution.

## Figures and Tables

**Table 1:** Biomass production of *C. balansae* and *C. striatus* seedlings under four treatments of N acquisition under two levels of P nutrition.

		Low Phosphate			
Biomass (g)	Plant sp.	−N−R	−N+R	+N−R	+N+R
Shoot	cba	0.165 ± 0.07 a	0.332 ± 0.07 b	0.485 ± 0.08 c	0.654 ± 0.03 d
	cst	0.312 ± 0.04 a	0.367 ± 0.01 b	0.645 ± 0.10 c	0.640 ± 0.05 c
Root	cba	0.050 ± 0.04 a	0.114 ± 0.008 b	0.189 ± 0.04 b	0.202 ± 0.008 c
	cst	0.115±0.02 a	0.141 ± 0.008 b	0.160 ± 0.02 c	0.186 ± 0.008 c
Nodules	cba	Θ	0.0279 ± 0.001 a	Θ	0.036 ± 0.005 a
	cst	Θ	0.0216 ± 0.003 a	Θ	0.031 ± 0.001 a
Whole plant	cba	0.215 ± 0.05 a	0.446 ± 0.03 b	0.674 ± 0.06 b	0.856 ± 0.01 c
	cst	0.327 ± 0.03 a	0.508 ± 0.04 b	0.805 ± 0.05 c	0.826 ± 0.06 c
		**High Phosphate**			
Shoot	cba	0.213 ± 0.04 a	0.403 ± 0.03 b	0.882 ± 0.07 d	0.828 ± 0.03 c
	cst	0.124 ± 0.01 a	0.566 ± 0.02 b	0.985 ± 0.06 d	0.623 ± 0.06 c
Root	cba	0.069 ± 0.006 a	0.124 ± 0.01 b	0.129 ± 0.03 b	0.142 ± 0.05 b
	cst	0.217 ± 0.007 a	0.173 ± 0.01 b	0.191 ± 0.04 c	0.136 ± 0.02 b
Nodules	cba	Θ	0.011 ± 0.003 a	Θ	0.010 ± 0.001 a
	cst	Θ	0.014 ± 0.001 a	Θ	0.012 ± 0.01 a
Whole plant	cba	0.329 ± 0.03 a	0.546 ± 0.01 b	1.49 ± 0.07 d	0.902 ± 0.01 c
	cst	0.241 ± 0.01 a	0.733 ± 0.06 b	0.931 ± 0.08 d	0.822 ± 0.07 c

Values are means ± standard deviation. Different letters indicate significant differences amongst treatments (*p* < 0.05). All means are values obtained from 10 plants, except for treatment −N−R where only 5 seedlings survived. Θ indicates no nodulation.

**Table 2:** Leaf area, leaf area:plant dry weight and photosynthetic rate of seedlings of *C. balansae* and *C. striatus* under four treatments of N acquisition under two levels of P nutrition.

		Low Phosphate			
	Plant sp.	−N−R	+N−R	−N+R	+N+R
Leaf area (cm^2^)	cba	0.703 ± 0.07 a	2.063 ± 0.06 a	2.067 ± 0.14 a	2.125 ± 0.10 a
	cst	0.986 ± 0.08 a	1.999 ± 0.08 a	2.097 ± 0.05 a	2.130 ± 0.12 a
Leaf area/DW	cba	1.954 ± 0.11 a	1.605 ± 0.20 b	2.432 ± 0.08 b	2.035 ± 0.05 b
	cst	3.520 ± 0.21 a	1.495 ± 0.25 b	3.616 ± 0.25 b	1.836 ± 0.06 b
Photosynthetic rate (μmol CO^2^·m^−2^·s^−1^)	cba	1.386 ± 0.13 a	2.717 ± 0.13 b	3.062 ± 0.09 b	3.448 ± 0.07 b
	cst	1.469 ± 0.16 a	2.924 ± 0.12 b	3.435 ± 0.17 b	3.848 ± 0.14 b
		**High Phosphate**			
Leaf area	cba	2.104 ± 0.13 a	2.045 ± 0.1 a	2.111 ± 0.10 a	2.057 ± 0.05 a
	cst	2.083 ± 0.03 a	1.089 ± 0.06 a	2.068 ± 0.02 a	2.143 ± 0.03 a
Leaf area/DW	cba	4.178 ± 0.08 a	1,062 ± 0.01 b	2.426 ± 0.13 b	2.007 ± 0.40 b
	cst	5.786 ± 0.13 a	1.061 ± 0.06 b	3.132 ± 0.15 b	1.514 ± 0.33 b
Photosynthetic rate (μmol CO^2^·m^−2^·s^−1^)	cba	2.786 ± 0.16 a	4.303 ± 0.15 b	3.503 ± 0.06 b	4.538 ± 0.14 b
	cst	2.717 ± 0.17 a	3.683 ± 0.13 b	3.269 ± 0.18 b	3.752 ± 0.07 b

Values are means ± standard deviation. Different letters indicate significant differences amongst treatments (*p* < 0.05). All means are values obtained from 10 plants, except for treatment −N−R where only 5 seedlings survived.
